# Anterior cruciate ligament transection alters the n-3/n-6 fatty acid balance in the lapine infrapatellar fat pad

**DOI:** 10.1186/s12944-019-1008-5

**Published:** 2019-03-18

**Authors:** Anne-Mari Mustonen, Reijo Käkelä, Mikko A. J. Finnilä, Andrew Sawatsky, Rami K. Korhonen, Simo Saarakkala, Walter Herzog, Tommi Paakkonen, Petteri Nieminen

**Affiliations:** 1 Institute of Biomedicine/Anatomy, School of Medicine, Faculty of Health Sciences, University of Eastern Finland, Kuopio, Finland; 2 Department of Environmental and Biological Sciences, Faculty of Science and Forestry, University of Eastern Finland, Joensuu, Finland; 3 Molecular and Integrative Biosciences Research Programme, Faculty of Biological and Environmental Sciences, University of Helsinki, Helsinki, Finland; 4 Helsinki University Lipidomics Unit (HiLIPID), Helsinki Institute for Life Science (HiLIFE), University of Helsinki, Helsinki, Finland; 5 Department of Applied Physics, Faculty of Science and Forestry, University of Eastern Finland, Kuopio, Finland; 6 Research Unit of Medical Imaging, Physics and Technology, Faculty of Medicine, University of Oulu, Oulu, Finland; 7 Medical Research Center Oulu, Oulu University Hospital and University of Oulu, Oulu, Finland; 8 Human Performance Laboratory, Faculty of Kinesiology, University of Calgary, Calgary, AB Canada; 9 Department of Diagnostic Radiology, Oulu University Hospital, Oulu, Finland

**Keywords:** Anterior cruciate ligament transection, Knee joint, N-3 polyunsaturated fatty acids, N-6 polyunsaturated fatty acids, *Oryctolagus cuniculus*, Osteoarthritis

## Abstract

**Background:**

The infrapatellar fat pad (IFP) of the knee joint has received lots of attention recently due to its emerging role in the pathogenesis of osteoarthritis (OA), where it displays an inflammatory phenotype. The aim of the present study was to examine the infrapatellar fatty acid (FA) composition in a rabbit (*Oryctolagus cuniculus*) model of early OA created by anterior cruciate ligament transection (ACLT).

**Methods:**

OA was induced randomly in the left or right knee joint of skeletally mature New Zealand White rabbits by ACLT, while the contralateral knee was left intact. A separate group of unoperated rabbits served as controls. The IFP of the ACLT, contralateral, and control knees were harvested following euthanasia 2 or 8 weeks post-ACLT and their FA composition was determined with gas chromatography–mass spectrometry.

**Results:**

The n-3/n-6 polyunsaturated FA (PUFA) ratio shifted in a pro-inflammatory direction after ACLT, already observed 2 weeks after the operation (0.20 ± 0.008 vs. 0.18 ± 0.009). At 8 weeks, the FA profile of the ACLT group was characterized with increased percentages of 20:4n-6 (0.44 ± 0.064 vs. 0.98 ± 0.339 mol-%) and 22:6n-3 (0.03 ± 0.014 vs. 0.07 ± 0.015 mol-%) and with decreased monounsaturated FA (MUFA) sums (37.19 ± 1.586 vs. 33.20 ± 1.068 mol-%) and n-3/n-6 PUFA ratios (0.20 ± 0.008 vs. 0.17 ± 0.008). The FA signature of the contralateral knees resembled that of the unoperated controls in most aspects, but had increased proportions of total n-3 PUFA and reduced MUFA sums.

**Conclusions:**

These findings provide novel information on the effects of early OA on the infrapatellar FA profile in the rabbit ACLT model. The reduction in the n-3/n-6 PUFA ratio of the IFP is in concordance with the inflammation and cartilage degradation in early OA and could contribute to disease pathogenesis.

**Electronic supplementary material:**

The online version of this article (10.1186/s12944-019-1008-5) contains supplementary material, which is available to authorized users.

## Background

Osteoarthritis (OA) is a degenerative joint disease and a major cause of pain and disability in elderly people [1]. It is characterized by the progressive degradation of articular cartilage, subchondral bone remodeling, and synovitis. The primary risk factors for developing OA are age, female gender, obesity, traumatic joint injury, and occupational joint loading [2]. Obesity has been assumed to be a predisposing factor for OA due to mechanical overload, but adipose tissue and immune cell infiltration may also contribute to OA pathogenesis by producing adipokines and cytokines [3, 4]. Generally, obesity is characterized by a systemic inflammatory state and an unbalanced polyunsaturated fatty acid (PUFA) profile in the body [5, 6]. Furthermore, an increase in the n-6/n-3 PUFA ratio in plasma is linked to increased knee pain and reduced function [7], which suggests potential implications in OA. On the other hand, anterior cruciate ligament (ACL) transection (ACLT) is a well-established surgical model of OA [8]. ACL injury can lead to the production of inflammatory mediators due to abnormal, traumatic cartilage loading contributing to the progression of OA [9].

The Hoffaʼs fat pad, or infrapatellar fat pad (IFP), has recently emerged as a source of inflammation in the knee OA [4]. It is an intracapsular but extrasynovial adipose tissue structure of the knee joint, where 98% of the fat consists of neutral lipids (mostly triacylglycerols, TAG) and 1% of phospholipids (PL) [10]. The IFP was previously believed to be mostly structural adipose tissue necessary for proper knee function, but recent data indicate that it is a metabolically active site of the joint that can affect the integrity of neighboring tissues [4]. The IFP can interact with synoviocytes and cartilage and induce both protective and disease-aggravating activities in OA. As an endocrine organ, it secretes adipokines, cytokines, fatty acids (FA), and PUFA-derived lipid mediators (LM). The IFP of OA patients has an inflammatory phenotype characterized by the secretion of adipokines and cytokines, and the composition of immune cells [3].

As FA display immunomodulatory properties, it has been hypothesized that their profiles in circulation and synovial fluid may be altered in OA, thereby contributing to disease progression [11]. N-3 and n-6 PUFA are important players in many disease states, as they are converted to different series of eicosanoids by cyclooxygenases (COX), lipoxygenases, and cytochrome P450 monooxygenases [12]. N-6 PUFA are precursors to pro-inflammatory LM, while n-3 PUFA produce less inflammatory or resolving LM. N-3 PUFA also partially replace 20:4n-6 from membrane PL and compete with n-6 PUFA for desaturases, elongases, and COX [13]. Increasing the amount of dietary n-3 PUFA can shift the balance of produced eicosanoids to a more beneficial direction [5]. N-3 PUFA can also suppress inflammation by regulating gene expression through interactions with nuclear receptors and transcription factors. Fish oils containing long-chain n-3 PUFA can be considered nutraceuticals with potentially beneficial effects on circulating lipid profiles [14].

The IFP has been demonstrated to be a source of FA and LM, such as the 20:4n-6-derived prostaglandin E_2_ (PGE_2_) [15]. FA can have an influence on cartilage, where 18:2n-6 has been documented to have pro-inflammatory effects via increased PGE_2_ production, while 16:0 and 18:1n-9 have been shown to inhibit cartilage degeneration and inflammation [11]. Similarly, n-3 PUFA, especially 20:5n-3, have anti-inflammatory and anti-destructive effects on cartilage [16, 17]. In vivo studies on the participation of FA in OA pathogenesis are scarce. Previously, FA profiles of synovial fluid have been compared between early and late-stage OA, and the levels of 10:0, 14:0, 17:0, and 18:1n-9 increased during disease progression [18]. In addition, the secretion of 20:4n-6 and thromboxane B_2_ was higher and that of lipoxin A_4_ lower from cultured IFP explants of OA patients compared to post-mortem donors without OA [15]. To further assess the significance of this emerging player in OA, the aim of the present study was to examine the influence of ACLT—a well-established surgical model that leads to the rapid development of early OA [19–21]—on the proportions of a comprehensive array of FA in the IFP of rabbits (*Oryctolagus cuniculus*) at two different time points following surgery. To the best of our knowledge, this is the first time the changes of FA composition in the IFP are characterized in the rabbit ACLT model. It was hypothesized that the proportions of pro-inflammatory FA would increase due to ACLT-induced early OA, whereas the more anti-inflammatory FA would decrease in the IFP.

## Methods

All experimental procedures were approved by the Animal Care Committee at the University of Calgary (#AC11–0035) and carried out according to the guidelines of the Canadian Council on Animal Care. Skeletally mature New Zealand White rabbits (strain 052 CR, *n* = 22 females, 12 months old, 4.8 ± 0.08 kg) were obtained from the Charles River Laboratories Inc. (Saint-Constant, QC, Canada). The rabbits were transported to the University of Calgary, where they were housed in single cages (76 × 64 × 41 cm) at 12 L:12D and ≈ 23 °C for 4 weeks before the experiment. The rabbits had free access to water and a pelleted maintenance diet (5326* Laboratory Rabbit Diet HF, LabDiet, St. Louis, MO, USA; http://www.labsupplytx.com/wp-content/uploads/2013/07/5326-Laboratory-Rabbit-Diet-HF.pdf).

Fifteen rabbits were anesthetized by established methods with subcutaneous (SC) acepromazine maleate (1 mg/kg; AceVet, Vétoquinol Inc., Lavaltrie, QC) and SC hydromorphone (0.15 mg/kg; HYDROmorphone Hydrochloride Injection USP, Sandoz Canada Inc., Boucherville, QC) and after 30 min they were placed under deep surgical anesthesia using 5% isoflurane (Fresenius Kabi Inc., Richmond Hill, ON, Canada) in medical oxygen (1 l/min). Surgical anesthesia was maintained with 1–2% isoflurane in medical oxygen. Heart rate and oxygen saturation were monitored with a SurgiVet pulse oximeter (Smiths Medical PM Inc., Waukesha, WI, USA) throughout the surgery. Unilateral ACLT was performed under aseptic laboratory conditions, randomly either on the left or right knee joint to avoid any possible bias caused by consistently choosing the same side. A 2.5–3 cm incision was made through the skin on the lateral side of the knee joint from just above the patella to the tibial plateau. A deeper incision went through the joint capsule about 2 mm posterior to the patella and the patellar ligament. The patella was then dislocated to allow visualization of the ACL. A hooked probe was placed around the ACL, which was cut with a number 12 scalpel. Full ACLT was confirmed visually and by performing the anterior drawer test. The joint capsule was sutured with a 4–0 interrupted silk suture. The wound was closed with a simple continuous suture using 3–0 Vicryl followed by a mattress suture on the surface. The contralateral knee was left intact. The procedure was conducted at 06.00–12.00 h, and the rabbits recovered post-operatively on a heating pad covered with a blanket until they were mobile and returned to their cages. The rabbits received SC buprenorphine (0.02 mg/kg; Vetergesic Multidose, Sogeval UK Ltd., York, UK) twice daily for a minimum of 2 days for pain relief. The health of the animals was closely monitored post-operatively and all efforts were made to minimize suffering. The left and right knees from a separate group of unoperated rabbits served as controls (*n* = 7, *n* of joints = 14).

After 2 or 8 weeks, the animals were anesthetized with isoflurane, as described above, subsequently euthanized with an intracardiac injection of pentobarbital sodium (200 mg/kg; Euthanyl, Bimeda-MTC Animal Health Inc., Cambridge, ON), and sampled at 07.00–12.00 h. The knees were dissected 2–3 cm above and below the knee joint, and the muscles were removed allowing clear visualization of all ligaments. The IFP was dissected with scissors behind the patellar ligament, placed in a 1.5 ml sample tube, snap-frozen in liquid nitrogen, and stored at −80 °C. The numbers of control, ACLT, and contralateral samples were 6, 8, and 8 at 2 weeks and 8, 7, and 7 at 8 weeks post-ACLT, respectively. Samples were transported on dry ice to Finland for further processing. The same experimental animals were previously reported to show signs of early OA [22].

For the FA analyses, subsamples of the IFP were transmethylated in methanolic H_2_SO_4_ under nitrogen atmosphere [23] and the formed FA methyl esters (FAME) were extracted with hexane and analyzed by a Shimadzu GC-2010 Plus gas chromatograph (Shimadzu, Kyoto, Japan) equipped with an auto injector, a flame ionization detector (FID), and ZB-wax capillary columns (Phenomenex, Torrance, CA, USA). The identities of the FAME structures were confirmed by using electron impact mass spectra recorded by a Shimadzu GCMS-QP2010 Ultra with a mass selective detector. The resulting chromatographic peaks from FID were manually integrated with the GCsolution software (*v*2.41.00) by Shimadzu. The results are represented as the FA composition (mol-%) of the IFP total lipids. The product/precursor ratios of n-3 and n-6 PUFA were calculated as follows: (20:5n-3 + 22:6n-3)/18:3n-3 and 20:4n-6/18:2n-6.

Statistical comparisons of FA profiles between the study groups were performed using a generalized linear model (IBM SPSS *v*21.0 software, IBM, Armonk, NY, USA). The model was performed with normal probability distribution of the residuals. The examined parameter was selected as the dependent variable, the experimental group as the model factor, and time as a covariate. The model included both experimental group and time as main effects as well as the time × group interaction. Changes in body mass were tested using paired samples *t*-tests. The *p* value < 0.05 was considered statistically significant. The results are presented as the mean ± SE.

## Results

**Fig. 1:**
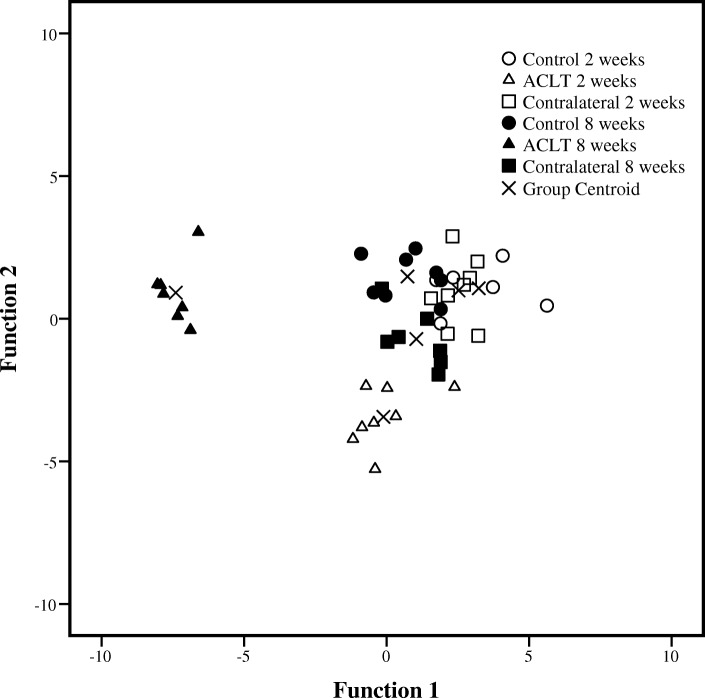
Discriminant analysis depicting the classification of fatty acid signatures of the rabbit infrapatellar fat pad in different study groups based on discriminant functions 1 and 2. ACLT anterior cruciate ligament transection

In the discriminant analysis, the 2- and 8-week ACLT groups were classified separately from each other, and separately from their corresponding control and contralateral knees, the latter of which were all grouped together based on the IFP FA composition (Fig. 1). The primary FA separating the groups included 16:1n-9, 22:6n-3, 22:5n-3, 20:4n-6, 20:3n-6, and 20:5n-3 (for function 1) and 18:2n-6 (for function 2). The analysis classified 100% of the samples correctly based on the study group.

**Fig. 2:**
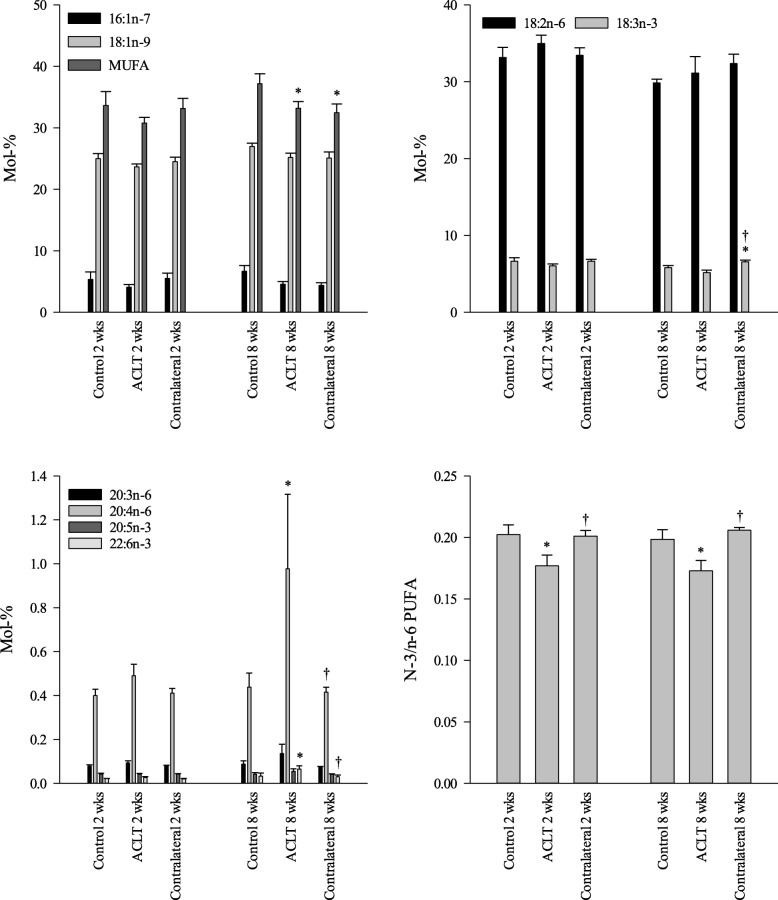
The percentages (mol-%) of selected monounsaturated fatty acids (MUFA) and polyunsaturated FA (PUFA) and the n-3/n-6 PUFA ratios in the rabbit infrapatellar fat pad in different study groups (mean + SE). ACLT anterior cruciate ligament transection, * differs from the control knee at the same time point, † differs from the ACLT knee at the same time point (generalized linear model, *p* < 0.05)

**Fig. 3:**
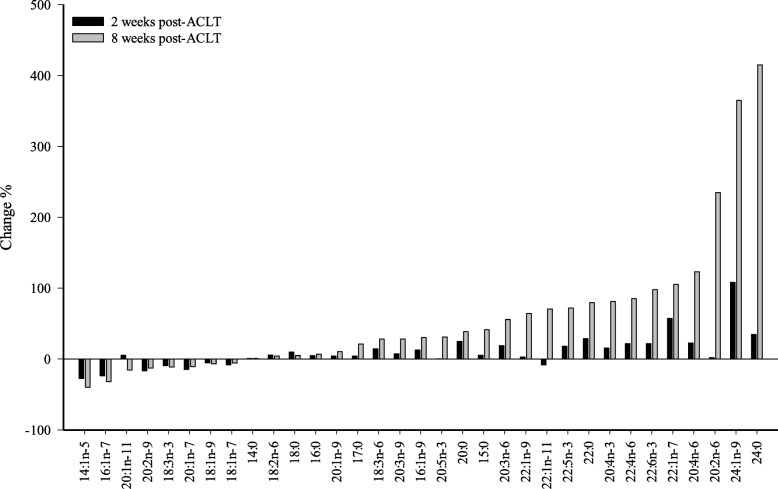
The relative changes (%) in the proportions of selected fatty acids (FA) in the infrapatellar fat pad of rabbits 2 and 8 weeks after anterior cruciate ligament transection (ACLT) compared to controls at the same time points. Negative values indicate that a FA decreased in proportion due to ACLT and positive values indicate its increase, the values calculated as (average mol-% in ACLT–average mol-% in control)/average mol-% in control

Regarding individual FA 2 weeks post-ACLT, the IFP of the ACLT group had higher proportions of 16:1n-9, C20–22 saturated FA (SFA), and 24:1n-9, lower percentages of 17:1n-8, and lower n-3/n-6 PUFA ratios than the controls (Figs. 2 and 3; Additional file 1: Table S1). The FA profiles in the contralateral knees were similar to those obtained for the control group knees, but differed from the ACLT group as follows: proportions of 17:1n-8 and ratios of n-3/n-6 PUFA were lower in the ACLT group and percentages of 18:3n-6, C20–24 SFA, 20:1n-9, 22:1n-7, 22:4n-6, and 24:1n-9 were higher.

After 8 weeks, the IFP of the ACLT group had higher proportions of 15:0, 16:1n-9, 17:0*i*, 17:0*ai*, 18:3n-6, C20–24 SFA, 20:4n-6, 20:4n-3, 22:1n-9, 22:4n-6, 22:5n-3, 22:6n-3, and 24:1n-9 than the controls, and lower percentages of total monounsaturated FA (MUFA) and lower n-3/n-6 PUFA ratios (Figs. 2 and 3; Additional file 1: Table S1). The percentages were higher for 18:3n-3 and total n-3 PUFA and lower for 17:1n-8, 20:1n-7, and total MUFA in the contralateral knees than in the controls. The ACLT knees showed higher proportions of 15:0, 16:1n-9, 17:0*i*, 17:0*ai*, C20–24 SFA, 20:1n-9, 20:4n-6, 20:4n-3, 22:4n-6, 22:5n-3, 22:6n-3, and most C22–24 MUFA and higher n-3 and n-6 PUFA product/precursor ratios compared to the contralateral group, whereas the percentages of 18:3n-3 and total n-3 PUFA, as well as ratios of n-3/n-6 PUFA were lower.

Comparing the 2-week and 8-week animals, there was an increase in the IFP proportions of 16:1n-9, 18:1n-9, 19:1n-8, 20:0, 20:4n-3, 24:0, 22:6n-3, and product/precursor ratios for n-3 PUFA and a reduction for 18:2n-6, 18:3n-3, n-6 PUFA, n-3 PUFA, and total PUFA from 2 weeks to 8 weeks, mostly regarding the ACLT groups (Fig. 2; Additional file 1: Table S1). Significant time × group interaction was observed for 16:1n-9, 17:0*i*, 17:1n-8, and 24:0 (Additional file 1: Table S1).

The operated rabbits had a slightly negative energy balance and had lost 8.7 ± 1.30% of their body mass 2 weeks after the operation and 8.0 ± 2.71% at 8 weeks compared to the initial body mass (paired *t*-test, *p* < 0.05).

## Discussion

The effects of early-stage OA on FA composition of the IFP were examined in the rabbit ACLT model, 2 and 8 weeks after surgical intervention. The main findings of this study were that *i*) ACLT changed the infrapatellar n-3/n-6 PUFA ratio towards a pro-inflammatory phenotype, *ii*) the FA profile of the IFP was altered as early as 2 weeks post-ACLT, *iii*) the ACLT-induced alterations in the proportions of particular FA became more pronounced at 8 weeks, and *iv*) the FA signature of the contralateral knee resembled that of the unoperated control in most aspects, but with some noteworthy exceptions.

The observed reduction in the n-3/n-6 PUFA ratio of the IFP may contribute to the inflammation and cartilage degradation in early OA. It is also in concordance with our data showing increased mRNA expression of inflammatory and cartilage-degrading factors interleukin IL-6, matrix metalloproteinase MMP-3, and MMP-13 in articular cartilage of similarly-treated rabbits at 2 weeks post-ACLT [24]. As an extrasynovial organ, the IFP does not directly interact with cartilage, but it has been proposed to be a source of adipokines, cytokines, FA, and LM that could contribute to the pathophysiological processes in OA [3, 15]. Other health issues that are associated with OA include obesity and low-grade systemic inflammation [2, 25]. Obesity is also linked to altered dietary and, consequently, body n-3/n-6 PUFA balance that has potential implications in OA [6, 7]. The n-3/n-6 PUFA ratio is of importance, as n-6 PUFA increase COX-2 protein levels and PGE_2_ production in chondrocytes [11, 26]. The increased proportion of 20:4n-6 in the IFP of the ACLT group is in line with earlier findings of Gierman et al., who observed that the secretion of 20:4n-6 was higher from the IFP of OA patients compared to post-mortem donors without OA [15].

In contrast, n-3 PUFA are metabolized into less inflammatory or pro-resolving LM [12, 27], and have anti-destructive effects on cartilage [16, 17]. In chondrocytes, n-3 PUFA, with 20:5n-3 being the most effective, reduce expression of COX-2, IL-1*α* and IL-1*β*, tumor necrosis factor-*α*, aggrecanases ADAMTS4–5, MMP-3, and MMP-13 [16]. In addition, n-3 PUFA may participate in bone remodeling by favoring osteoblastogenesis [28]. The elevated 22:6n-3 percentage in the ACLT knees of the rabbits is similar to earlier findings from OA patients [15]. As the IFP has been shown to induce both protective and disease-aggravating activities in OA [4], pro-resolving LM, such as resolvins, protectins, and maresins, derived from 22:6n-3 could potentially contribute to the resolution pathways that are activated in OA [27, 29]. In addition to joint health, long-chain n-3 PUFA can have beneficial effects on dyslipidaemia and the cardiovascular system [14].

The FA profile of the IFP was already altered 2 weeks post-ACLT. The affected FA were generally of minor proportions, and the most interesting change was the reduction in the n-3/n-6 PUFA ratio. According to previous findings in the rabbit model, the first signs of OA have typically been documented 4 weeks post-ACLT [21, 30–32]. These include reduced proteoglycan (PG) content, fibrillation, and lowered biomechanical stiffness [20, 33]. Bone mineral density has been shown to be decreased and blood flow increased in periarticular bone at 2 weeks post-ACLT [34]. Ojanen et al. reported a loss of fixed charge density from articular cartilage 2 weeks post-ACLT [22]. The FA signature in adipose tissue/plasma is known to change rapidly in response to various stimuli, for instance, fasting and dietary changes [35, 36]. Against this background, the altered FA profiles after 2 weeks of intervention were expected and could represent early responses to inflammation and tissue damage. This could also be connected to the reduced fixed charge density of PG reported previously [22].

As shown in Fig. 3, ACLT reduced the relative proportions of 16:1n-7, 18:3n-3, and C18 MUFA at 8 weeks post-ACLT, while percentages of longer-chain SFA and MUFA tended to increase simultaneously with several C20–22 PUFA. Many of these changes emerged at 2 weeks already and would be consistent with a situation where cellular membranes, and FA abundant in their PL pool, had increased in proportion at the expense of TAG residing in storage lipid droplets. For example, adipocyte sphingomyelin is rich in long-chain SFA and MUFA [37]. The overall pattern of FA modifications observed in the rabbits is not unique to this model, as similar changes have been documented in adipose tissue during food deprivation of rodents [36], supporting the present FA results in the rabbits with a slightly negative energy balance compared to the initial body mass. The IFP has been assumed to be resistant to starvation [4], but recent data have shown that its volume can decrease due to weight loss [38]. The effects of OA on the composition of the IFP have not been studied systematically. However, it is known that OA can increase vascularization, inflammatory infiltration, and thickness of the interlobular septa, i.e., fibrosis [39]. Moreover, OA patients with IFP lymphocytic infiltration have thicker lobuli septa and smaller adipose lobuli than OA patients without lymphocytic infiltration. These adaptations may contribute to the observed changes in the infrapatellar FA composition after ACLT.

There were some notable differences in the FA profiles between the contralateral and control knees 8 weeks post-ACLT. The contralateral knees are often used as within-animal controls in ACLT studies [30, 31, 33]. However, altered post-operative loading patterns may affect cartilage and bone in the contralateral joint [34, 40]. Such changes have been documented, for instance, in dynamic elastic modulus, PG concentration, collagen content and orientation angle, permeability, and fibril network modulus of articular cartilage between the control and contralateral knees in this animal model [21, 32]. When compared to the unoperated control knee, the present study revealed an increase in the n-3 PUFA sum and a decrease in the MUFA sum, findings that are not trivial to explain. One possibility is that the systemic redistribution of circulation following the operation, and the consequent uneven flow of metabolites to the limbs, may have affected the tissues of both the operated and the contralateral knee. This might involve the neural regulation of circulation, but inflammatory signaling and edema in the operated knee could also have metabolic reflections in other parts of the body that would not be present in the unoperated controls. In addition, the distribution of n-3 PUFA to the peripheral circulation could be used to induce the resolution phase of inflammation, which employs n-3 PUFA-derived LM, such as resolvins. The previously described changes in gait and muscle use in the contralateral knee of OA patients emphasize the importance of treating the contralateral knee as an anatomical site affected by the diseased joint [41]. Even though the FA signature of the contralateral knee remained similar to that of the unoperated control in most aspects, its use as an unaffected control for studying the FA profile of the IFP cannot be recommended.

The proportion of total MUFA was also reduced in the ACLT knees 8 weeks post-ACLT. The possible role of MUFA in OA is not clear, but 18:1n-9 has exerted anti-destructive and anti-inflammatory effects on chondrocytes and cartilage in vitro [11]. The present findings are in line with this notion, as the proportion of total MUFA was reduced post-ACLT even though the decreasing trend in 18:1n-9, the most abundant individual MUFA, did not reach significance. Previously, 18:1n-9 has been identified as a critical metabolite for discriminating between early and late-stage OA, with increased levels in synovial fluid during disease progression [18]. Regarding SFA, 16:0 has been documented to inhibit cartilage destruction in vitro [11], but the present study did not find any effects of ACLT on the proportions of major SFA.

There are some limitations in the present study. The control group numbers were relatively small, but similar numbers of rabbits/joints have been used successfully in previous publications [21]. Based on earlier studies, OA changes in this model are systematic, and a relatively small number of samples has yielded statistical differences in primary outcome variables. For statistical comparison, the minimum number of joints in each group would be 6, which was attained. To reduce the number of experimental animals, both knee joints were used from the control group animals, as has been done previously [21, 32]. The contralateral knees were not sham-operated to avoid infection and to make the present study comparable to earlier ones. Unfortunately, the food intake of the animals was not recorded during the study. For this reason, it is not known, whether there were differences in the energy balance or in the intake of essential PUFA precursors between the study groups. However, since all groups received the same diet, dietary effects on the observed differences in FA profiles can be assumed to be minor. Another limitation was that the FA composition was not determined from separate neutral and PL fractions but from total lipids and, thus, it mostly reflected the IFP storage fats rather than the membrane lipids.

The progression of OA in the rabbit ACLT model is rapid [8]. Early stages of secondary OA can develop within weeks and, thus, the selected model served well to address the aims of this study. Smaller species, such as rats and mice, have knee joint surfaces that are too small to allow for cell deformation experiments that were conducted in these animals. Our choice of rabbits allows for comparisons with earlier studies with the same surgical protocol. The present study provides novel information about the role of the IFP in inflammation, which has relevance in the progression of OA [4]. Studying the normal and abnormal loading of the knee joint, and the patellofemoral and tibiofemoral joints, is not possible in cell cultures or explants. The rabbit knee differs in anatomy and size of intra-articular structures, as well as in the range of motion from the human knee [42]. These differences have to be considered when translating the present results to the context of early OA in humans. Nevertheless, the basic bony, ligamentous, and muscular structures are similar [42, 43], and OA development, albeit much faster in rabbits than in humans, includes a similar array of pathological events [8]. Thus, the FA phenomena described here could have relevance in the development of human OA.

## Conclusions

The IFP had a more pro-inflammatory lipid profile in the ACLT rabbits compared to the controls as early as 2 weeks post-ACLT. The altered n-3/n-6 PUFA balance suggests the involvement of the IFP in the inflammatory processes of OA in the knee. In addition, the divergence of the contralateral knee from the control knee FA profile indicates that it is per se an interesting research target. Even though the FA signature of the contralateral knee remained similar to that of the unoperated control joint in most aspects, its use as an unaffected within-animal control for studying the FA profiles of the IFP cannot be recommended without caution. The ACLT-induced reduction in the n-3/n-6 PUFA ratio of the IFP is in concordance with the inflammation and cartilage degradation in early OA. It may be a novel contributing factor and, thus, a possible therapeutic target, in the study and treatment of disease pathogenesis.

## Additional file

Additional file 1: **Table S1.** The percentages (mol-%) of fatty acids in the rabbit infrapatellar fat pads in different study groups (mean ± SE). Means with similar superscript letters are significantly different from each other (generalized linear model, *p* < 0.05). (DOCX 27 kb)

## Abbreviations

ACL: Anterior cruciate ligament
ACLT: Anterior cruciate ligament transection
*ai*: Anteiso
COX: Cyclooxygenase
FA: Fatty acid
FAME: Fatty acid methyl ester
FID: Flame ionization detector
*i*: Iso
IFP: Infrapatellar fat pad
IL: Interleukin
LM: Lipid mediator
MMP: Matrix metalloproteinase
MUFA: Monounsaturated fatty acid
OA: Osteoarthritis
PG: Proteoglycan
PGE_2_: Prostaglandin E_2_
PL: Phospholipid
PUFA: Polyunsaturated fatty acid
SC: Subcutaneous
SFA: Saturated fatty acid
TAG: Triacylglycerol
UFA: Unsaturated fatty acid
