# Supplementary material for: Anterior cruciate ligament transection alters the n-3/n-6 fatty acid balance in the lapine infrapatellar fat pad
Source: Lipids Health Dis. 2019 Mar 18;18:67. doi: 10.1186/s12944-019-1008-5 (PMC6421636; doi:10.1186/s12944-019-1008-5)
Supplement: Supplementary file 1 — Table S1. The percentages (mol-%) of fatty acids in the rabbit infrapatellar fat pads in different study groups (mean ± SE). Means with similar superscript letters are significantly different from each other (generalized linear model, p < 0.05). (DOCX 27 kb) [file 12944_2019_1008_MOESM1_ESM.docx]

**Table S1.** The percentages (mol-%) of fatty acids in the rabbit infrapatellar fat pads in different study groups (mean ± SE). Means with similar superscript letters are significantly different from each other (generalized linear model, *p*<0.05).

|  | Control 2 wks | ACLT 2 wks | Contralateral 2 wks | Control 8 wks | ACLT 8 wks | Contralateral 8 wks | *p time* | *p group* | *p interaction* |
| --- | --- | --- | --- | --- | --- | --- | --- | --- | --- |
| Number of fat pads | 6 | 8 | 8 | 8 | 7 | 7 |  |  |  |
| 14:0 | 1.205 ± 0.060 | 1.213 ± 0.067 | 1.238 ± 0.059 | 1.272 ± 0.037 | 1.282 ± 0.111 | 1.177 ± 0.048 | 0.622 | 0.796 | 0.487 |
| 14:1n-5 | 0.268 ± 0.083 | 0.195 ± 0.027 | 0.265 ± 0.054 | 0.355 ± 0.058 | 0.214 ± 0.033 | 0.192 ± 0.018 | 0.765 | 0.051 | 0.221 |
| 15:0 | 0.413 ± 0.030 | 0.435 ± 0.020 | 0.423 ± 0.025 | 0.345 ± 0.016^A^ | 0.488 ± 0.041^AB^ | 0.410 ± 0.025^B^ | 0.633 | 0.004 | 0.053 |
| 16:0 | 16.892 ± 0.513 | 17.696 ± 0.643 | 17.461 ± 0.654 | 17.435 ± 0.629 | 18.624 ± 1.208 | 17.647 ± 0.527 | 0.324 | 0.345 | 0.860 |
| 16:1n-9 | 0.261 ± 0.005^A^ | 0.294 ± 0.016^AD^ | 0.276 ± 0.010 | 0.271 ± 0.010^B^ | 0.354 ± 0.024^BCD^ | 0.277 ± 0.008^C^ | 0.021 | 0.000008 | 0.042 |
| 16:1n-7 | 5.358 ± 1.212 | 4.094 ± 0.433 | 5.513 ± 0.848 | 6.701 ± 0.890 | 4.569 ± 0.450 | 4.383 ± 0.431 | 0.687 | 0.051 | 0.200 |
| 16:1n-5 | 0.039 ± 0.008 | 0.030 ± 0.002 | 0.039 ± 0.006 | 0.045 ± 0.005 | 0.039 ± 0.005 | 0.033 ± 0.003 | 0.492 | 0.242 | 0.280 |
| 17:0*i* | 0.039 ± 0.002 | 0.035 ± 0.002 | 0.037 ± 0.002 | 0.034 ± 0.002^A^ | 0.049 ± 0.003^AB^ | 0.035 ± 0.002^B^ | 0.117 | 0.004 | 0.000003 |
| 17:0*ai* | 0.170 ± 0.007 | 0.188 ± 0.015 | 0.184 ± 0.015 | 0.167 ± 0.008^A^ | 0.234 ± 0.018^AB^ | 0.181 ± 0.011^B^ | 0.181 | 0.002 | 0.076 |
| 17:0 | 0.584 ± 0.066 | 0.610 ± 0.042 | 0.560 ± 0.041 | 0.492 ± 0.037 | 0.596 ± 0.032 | 0.615 ± 0.032 | 0.592 | 0.229 | 0.170 |
| 17:1n-8 | 0.445 ± 0.027^A^ | 0.362 ± 0.014^AB^ | 0.433 ± 0.025^B^ | 0.433 ± 0.019^C^ | 0.416 ± 0.018 | 0.382 ± 0.018^C^ | 0.848 | 0.035 | 0.018 |
| 18:0 | 5.785 ± 0.655 | 6.354 ± 0.433 | 5.475 ± 0.424 | 5.828 ± 0.544 | 6.114 ± 0.534 | 7.050 ± 0.467 | 0.234 | 0.567 | 0.113 |
| 18:1n-9 | 24.996 ± 0.816^A^ | 23.637 ± 0.498^B^ | 24.486 ± 0.738 | 26.966 ± 0.544^A^ | 25.191 ± 0.682^B^ | 25.089 ± 1.001 | 0.012 | 0.054 | 0.578 |
| 18:1n-7 | 1.840 ± 0.140 | 1.690 ± 0.045 | 1.720 ± 0.089 | 1.938 ± 0.117 | 1.828 ± 0.075 | 1.705 ± 0.064 | 0.292 | 0.107 | 0.638 |
| 18:1n-5 | 0.074 ± 0.011 | 0.069 ± 0.006 | 0.073 ± 0.007 | 0.092 ± 0.005 | 0.076 ± 0.005 | 0.075 ± 0.006 | 0.076 | 0.226 | 0.445 |
| 18:2n-7 | 0.122 ± 0.004 | 0.118 ± 0.006 | 0.108 ± 0.006 | 0.115 ± 0.007 | 0.129 ± 0.009 | 0.125 ± 0.005 | 0.148 | 0.455 | 0.140 |
| 18:2n-6 | 33.166 ± 1.299^A^ | 34.987 ± 1.081 | 33.439 ± 0.978 | 29.842 ± 0.497^A^ | 31.135 ± 2.159 | 32.366 ± 1.218 | 0.004 | 0.356 | 0.447 |
| 18:3n-6 | 0.098 ± 0.011 | 0.112 ± 0.005^A^ | 0.095 ± 0.006^A^ | 0.095 ± 0.005^B^ | 0.122 ± 0.009^B^ | 0.105 ± 0.007 | 0.298 | 0.004 | 0.534 |
| 19:1n-8 | 0.065 ± 0.002 | 0.067 ± 0.005 | 0.066 ± 0.003 | 0.076 ± 0.007 | 0.089 ± 0.013 | 0.065 ± 0.004 | 0.034 | 0.131 | 0.220 |
| 18:3n-3 | 6.649 ± 0.438 | 6.040 ± 0.254^C^ | 6.648 ± 0.245 | 5.809 ± 0.280^A^ | 5.153 ± 0.324^BC^ | 6.571 ± 0.217^AB^ | 0.007 | 0.001 | 0.240 |
| 18:2*c*9*t*11 | 0.092 ± 0.008 | 0.080 ± 0.008 | 0.088 ± 0.008 | 0.081 ± 0.005 | 0.083 ± 0.007 | 0.079 ± 0.008 | 0.314 | 0.800 | 0.610 |
| 20:0 | 0.070 ± 0.002^A^ | 0.088 ± 0.006^AB^ | 0.063 ± 0.003^BE^ | 0.076 ± 0.006^C^ | 0.105 ± 0.010^CD^ | 0.076 ± 0.004^DE^ | 0.007 | 0.000001 | 0.566 |
| 20:1n-11 | 0.024 ± <0.001 | 0.026 ± 0.002 | 0.024 ± 0.001 | 0.026 ± 0.002 | 0.022 ± 0.001 | 0.022 ± 0.001 | 0.445 | 0.277 | 0.172 |
| 20:1n-9 | 0.200 ± 0.010 | 0.209 ± 0.011^A^ | 0.175 ± 0.006^A^ | 0.198 ± 0.012 | 0.219 ± 0.010^B^ | 0.177 ± 0.006^B^ | 0.658 | 0.000091 | 0.785 |
| 20:1n-7 | 0.015 ± 0.003 | 0.013 ± 0.001 | 0.012 ± 0.001 | 0.019 ± 0.003^A^ | 0.017 ± 0.003 | 0.011 ± 0.001^A^ | 0.091 | 0.017 | 0.330 |
| 20:2n-9 | 0.036 ± 0.001 | 0.030 ± 0.002 | 0.033 ± 0.002 | 0.041 ± 0.003 | 0.036 ± 0.006 | 0.032 ± 0.003 | 0.151 | 0.083 | 0.448 |
| 20:2n-6 | 0.022 ± 0.002 | 0.022 ± 0.002 | 0.019 ± 0.001 | 0.033 ± 0.009 | 0.110 ± 0.063 | 0.023 ± 0.003 | 0.071 | 0.106 | 0.131 |
| 20:3n-9 | 0.028 ± 0.002 | 0.030 ± 0.002 | 0.027 ± 0.001 | 0.030 ± 0.004 | 0.039 ± 0.010 | 0.025 ± 0.002 | 0.330 | 0.134 | 0.449 |
| 20:3n-6 | 0.079 ± 0.006 | 0.093 ± 0.010 | 0.079 ± 0.004 | 0.088 ± 0.015 | 0.137 ± 0.042 | 0.074 ± 0.003 | 0.255 | 0.054 | 0.349 |
| 20:4n-6 | 0.400 ± 0.028 | 0.491 ± 0.052 | 0.411 ± 0.021 | 0.438 ± 0.064^A^ | 0.977 ± 0.339^AB^ | 0.415 ± 0.023^B^ | 0.094 | 0.016 | 0.108 |
| 20:4n-3 | 0.016 ± 0.001 | 0.019 ± 0.001^C^ | 0.017 ± 0.001 | 0.020 ± 0.004^A^ | 0.037 ± 0.008^ABC^ | 0.021 ± 0.004^B^ | 0.007 | 0.025 | 0.121 |
| 20:5n-3 | 0.042 ± 0.004 | 0.042 ± 0.003 | 0.042 ± 0.002 | 0.042 ± 0.007 | 0.055 ± 0.011 | 0.041 ± 0.002 | 0.318 | 0.316 | 0.341 |
| 22:0 | 0.032 ± 0.003^A^ | 0.041 ± 0.004^AB^ | 0.031 ± 0.002^B^ | 0.034 ± 0.008^C^ | 0.061 ± 0.015^CD^ | 0.028 ± 0.003^D^ | 0.234 | 0.003 | 0.203 |
| 22:1n-11 | 0.022 ± 0.006 | 0.020 ± 0.003 | 0.017 ± 0.004 | 0.020 ± 0.005 | 0.033 ± 0.009^A^ | 0.013 ± 0.002^A^ | 0.547 | 0.041 | 0.131 |
| 22:1n-9 | 0.034 ± 0.008 | 0.035 ± 0.005 | 0.025 ± 0.004 | 0.033 ± 0.005^A^ | 0.054 ± 0.012^AB^ | 0.027 ± 0.003^B^ | 0.208 | 0.014 | 0.236 |
| 22:1n-7 | 0.003 ± 0.001 | 0.005 ± 0.001^A^ | 0.003 ± <0.001^A^ | 0.005 ± 0.002 | 0.009 ± 0.004 | 0.003 ± 0.001 | 0.119 | 0.037 | 0.561 |
| 22:4n-6 | 0.142 ± 0.016 | 0.173 ± 0.019^A^ | 0.133 ± 0.007^A^ | 0.166 ± 0.038^B^ | 0.307 ± 0.084^BC^ | 0.137 ± 0.009^C^ | 0.066 | 0.007 | 0.149 |
| 22:5n-3 | 0.150 ± 0.013 | 0.178 ± 0.021 | 0.145 ± 0.008 | 0.171 ± 0.037^A^ | 0.295 ± 0.072^AB^ | 0.144 ± 0.007^B^ | 0.082 | 0.009 | 0.140 |
| 24:0 | 0.015 ± 0.002 | 0.020 ± 0.003^AD^ | 0.014 ± 0.001^A^ | 0.022 ± 0.007^B^ | 0.115 ± 0.052^BCD^ | 0.024 ± 0.007^C^ | 0.016 | 0.013 | 0.034 |
| 22:6n-3 | 0.022 ± 0.001 | 0.027 ± 0.004^C^ | 0.021 ± 0.002 | 0.033 ± 0.014^A^ | 0.065 ± 0.015^ABC^ | 0.030 ± 0.008^B^ | 0.005 | 0.028 | 0.152 |
| 24:1n-9 | 0.011 ± 0.002^A^ | 0.023 ± 0.006^AB^ | 0.011 ± 0.002^B^ | 0.015 ± 0.005^C^ | 0.069 ± 0.038^CD^ | 0.013 ± 0.004^D^ | 0.132 | 0.021 | 0.196 |
| SFA | 25.204 ± 1.080 | 26.679 ± 0.699 | 25.486 ± 0.929 | 25.705 ± 1.209 | 27.668 ± 1.661 | 27.243 ± 0.698 | 0.192 | 0.244 | 0.824 |
| MUFA | 33.655 ± 2.248 | 30.768 ± 0.932 | 33.136 ± 1.667 | 37.193 ± 1.586^AB^ | 33.200 ± 1.068^A^ | 32.468 ± 1.397^B^ | 0.123 | 0.040 | 0.298 |
| PUFA | 41.062 ± 1.637^A^ | 42.440 ± 1.158 | 41.303 ± 1.207 | 37.004 ± 0.624^A^ | 38.679 ± 2.182 | 40.189 ± 1.402 | 0.005 | 0.363 | 0.457 |
| n-6 PUFA | 33.906 ± 1.282^A^ | 35.878 ± 1.114 | 34.174 ± 1.000 | 30.661 ± 0.554^A^ | 32.787 ± 2.014 | 33.120 ± 1.205 | 0.009 | 0.201 | 0.566 |
| n-3 PUFA | 6.880 ± 0.425 | 6.305 ± 0.233^C^ | 6.873 ± 0.247 | 6.076 ± 0.238^A^ | 5.605 ± 0.267^BC^ | 6.808 ± 0.205^AB^ | 0.010 | 0.001 | 0.274 |
| n-3/n-6 PUFA | 0.202 ± 0.008^A^ | 0.177 ± 0.009^AB^ | 0.201 ± 0.005^B^ | 0.198 ± 0.008^C^ | 0.173 ± 0.008^CD^ | 0.206 ± 0.002^D^ | 0.848 | 0.000009 | 0.747 |
| n-9 PUFA | 0.063 ± 0.002 | 0.059 ± 0.002 | 0.059 ± 0.002 | 0.071 ± 0.006 | 0.074 ± 0.012 | 0.057 ± 0.004 | 0.116 | 0.163 | 0.259 |
| UFA/SFA | 3.003 ± 0.182 | 2.763 ± 0.101 | 2.960 ± 0.153 | 2.954 ± 0.205 | 2.698 ± 0.282 | 2.681 ± 0.089 | 0.337 | 0.331 | 0.741 |
| Prod/prec n-6 PUFA | 0.012 ± 0.001 | 0.014 ± 0.001 | 0.012 ± <0.001 | 0.015 ± 0.002 | 0.034 ± 0.014^A^ | 0.013 ± 0.001^A^ | 0.069 | 0.048 | 0.114 |
| Prod/prec n-3 PUFA | 0.009 ± 0.001 | 0.011 ± 0.002^B^ | 0.009 ± <0.001 | 0.014 ± 0.005 | 0.025 ± 0.006^AB^ | 0.011 ± 0.002^A^ | 0.021 | 0.012 | 0.135 |

ACLT = anterior cruciate ligament transection, *i* = iso, *ai* = anteiso, *c* = cis, *t* = trans, SFA = saturated fatty acids, MUFA = monounsaturated fatty acids, PUFA = polyunsaturated fatty acids, UFA = MUFA + PUFA, prod = product, prec = precursor
